# Kounis syndrome secondary to amoxicillin/clavulanic acid administration: a case report and review of literature

**DOI:** 10.1186/s13104-015-1072-5

**Published:** 2015-03-26

**Authors:** Dissanayake Mudiyanselage Priyantha Udaya Kumara Ralapanawa, Senanayake Abeysinghe Mudiyanselage Kularatne

**Affiliations:** Department of Medicine, Consultant Physician & Senior Lecturer, University of Peradeniya, Peradeniya, Sri Lanka; Department of Medicine, Senior Professor in Medicine, Senior Consultant Physician, University of Peradeniya, Peradeniya, Sri Lanka

**Keywords:** Kounis syndrome, Amoxicillin/clavulanic acid, Acute coronary syndrome

## Abstract

**Background:**

Kounis syndrome is the concurrence of acute coronary syndromes with mast cells activation induced by hypersensitivity and anaphylactoid insults and is increasingly encountered in clinical practice. The main pathophysiological mechanism is vasospasm of the epicardial coronary arteries due to increased inflammatory mediators that are released during a hypersensitivity reaction.

**Case presentation:**

A 74-year -old Sinhalese man with diabetes mellitus was admitted with four day history of high fever with chills and rigors. His urine analysis and blood investigations revealed evidence of urinary tract infection. After excluding allergic conditions, he was given amoxicillin/clavulanic acid intravenously. About 20 minutes after the first dose he felt severe itching of body, nausea , dizziness and sever retrosternal chest pain. Urgent electrocardiogram was taken and it showed widespread ST segment elevations. He was treated for anaphylactic shock as well as acute coronary syndrome and was able to be discharged within a few days.

**Conclusion:**

This case highlights the occurrence of acute coronary syndrome following drug induced anaphylaxis. Acute coronary syndrome of this nature may be completely atypical and overlooked. Kounis syndrome should be borne in mind in the event of anaphylactic episode wherein the electrocardiogram becomes essential.

## Background

Kounis syndrome (allergic angina and allergic myocardial infarction) has been described as coincidental occurrence of acute coronary syndromes with conditions associated with mast cell activation, such as allergies or hypersensitivity and anaphylactoid insults [1,2]. It is caused by inflammatory mediators such as histamine, neutral proteases, arachidonic acid products such as leukotrienes, platelet activating factor and a variety of cytokines and chemokines released during the activation process [1]. There are several triggers that have been reported as capable of inducing Kounis syndrome by facilitating the release of various inflammatory mediators. These trigger may be drugs, foreign bodies, chemicals, environmental exposure, diseases or certain other conditions [3]. The cardiac involvement occurs in a considerable number of patients during episodes of anaphylaxis, and frequently in patients with prior coronary disease, although it has also been observed in patients with healthy coronary vessels. Vasospasm of the coronary arteries has been implicated as the main pathophysiologic mechanism [4]. The manifestations of acute coronary syndrome (ACS) in drug-induced hypersensitivity reactions may be completely atypical and overlooked. Kounis syndrome is a potentially life threatening event and delay in diagnosis and treatment will carry very poor prognosis.

## Case presentation

A 74-year-old Sinhalese man with diabetes mellitus and hyperlipidaemia was admitted to hospital with history of continuously high fever for four days duration. The fever was associated with chills and rigors. He had no myalgia but experienced nausea and had vomited twice before admission. He was a quite active before this illness and did not have any history of allergy. On clinical examination, he was ill looking and mildly dehydrated. His heart sounds were distinct with no additional sounds, and there were no abnormal signs in the other systems. The radial pulse rate was 72beats/min, regular and his blood pressure (BP) was 140/82 mmHg. Urine analysis showed 25-30 pus cell and 4-6 red blood cells (RBC) per high power field with a few organisms. His white cell count was 14.71×10^9^/l with 75% neutrophils. C-reactive protein was 179 mg/dl, random blood sugar was 98 mg/dl, serum sodium-142 meq/l, potassium-4.5 meq/l. He was treated for urinary tract infection with amoxicillin-clavulanic acid 1.2 grams intravenously.

**Figure 1:**
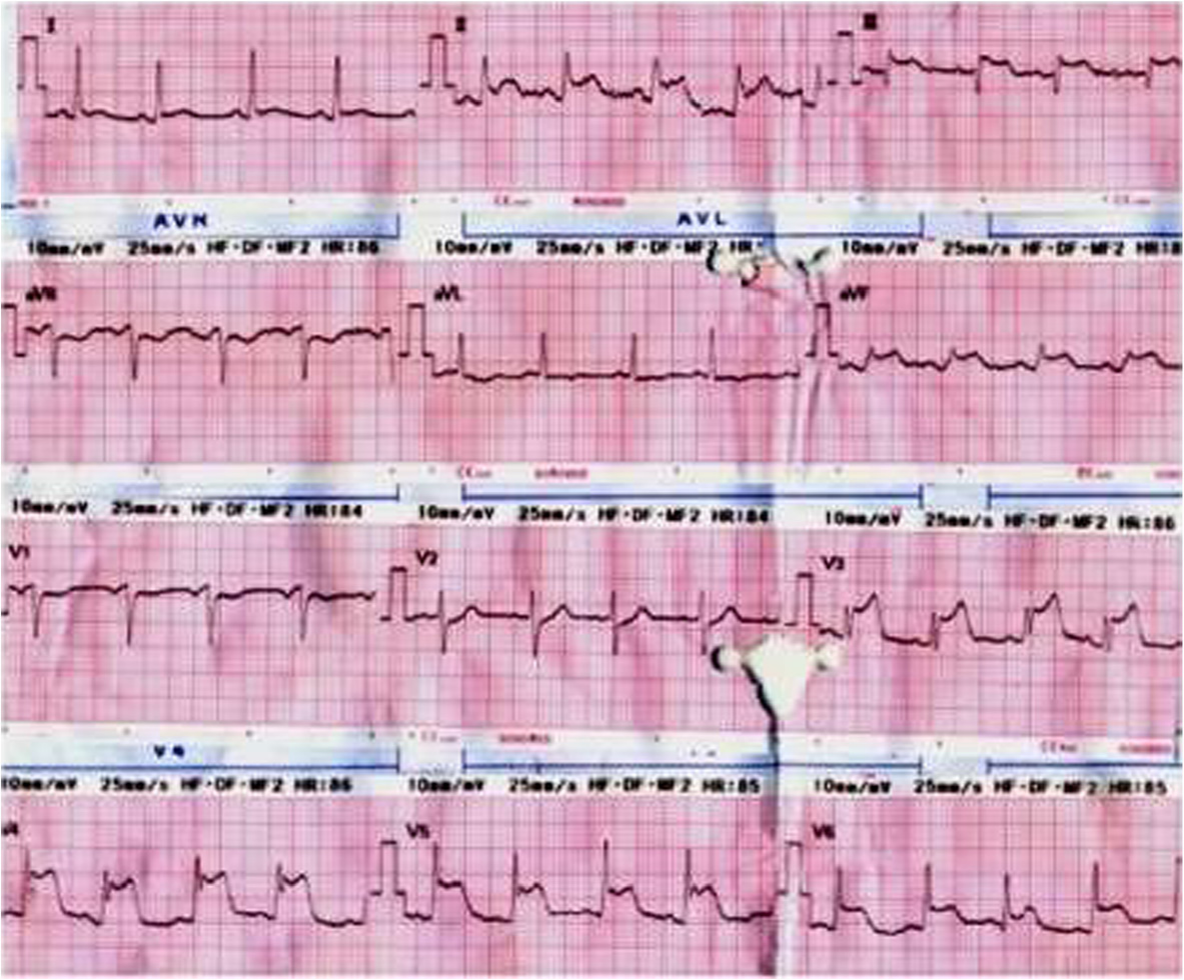
**Electrocardiogram taken at the time of chest pain (at 21.25 hours) showing ST-segment elevation in leads 11.111.aV1-3, F, V3-V6.**

Twenty minutes after administrating the drug, the patient exhibited a generalized maculopapular rash on his trunk and limbs, which was accompanied by intense itching. He felt sense of instability, palpitations, central chest tightness and excessive sweating. His BP was 80/60 mmHg; peripheral oxygen saturation(SpO2) was 95%. Urgent ECG was taken(Figure 1) showed ST elevation of 2 mm in leads 11,111,aVF,V3-V6. He was immediately treated for anaphylactic shock with 0.5 ml (1:1000) adrenalin intramuscularly (IM) Hydrocortisone 200 mg intravenously (IV) and chlorpheniramine 10 mg IV. He felt better and improved over the next 10 minutes. His rash was settling and blood pressure picked up to 102/78 mmHg. As electrocardiogram (ECG) shows widespread ST elevation aspirin 300 mg, clopidogrel 300 mg and atovastatin 40 mg stat doses were given. By this time his chest pain had settled.

**Figure 2:**
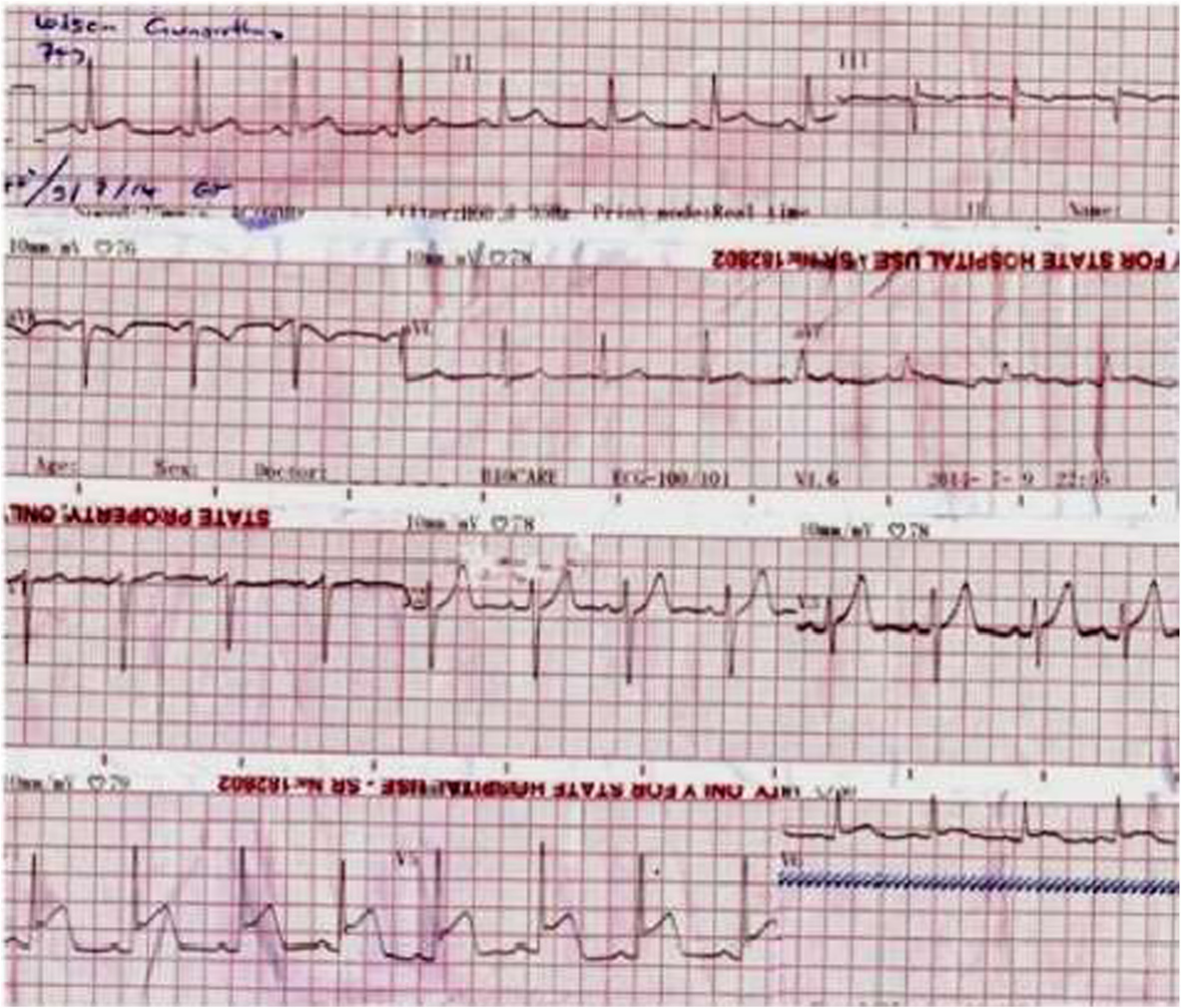
**Electrocardiogram taken at 22.40 hours showing resolving ST segment elevation.**

**Figure 3:**
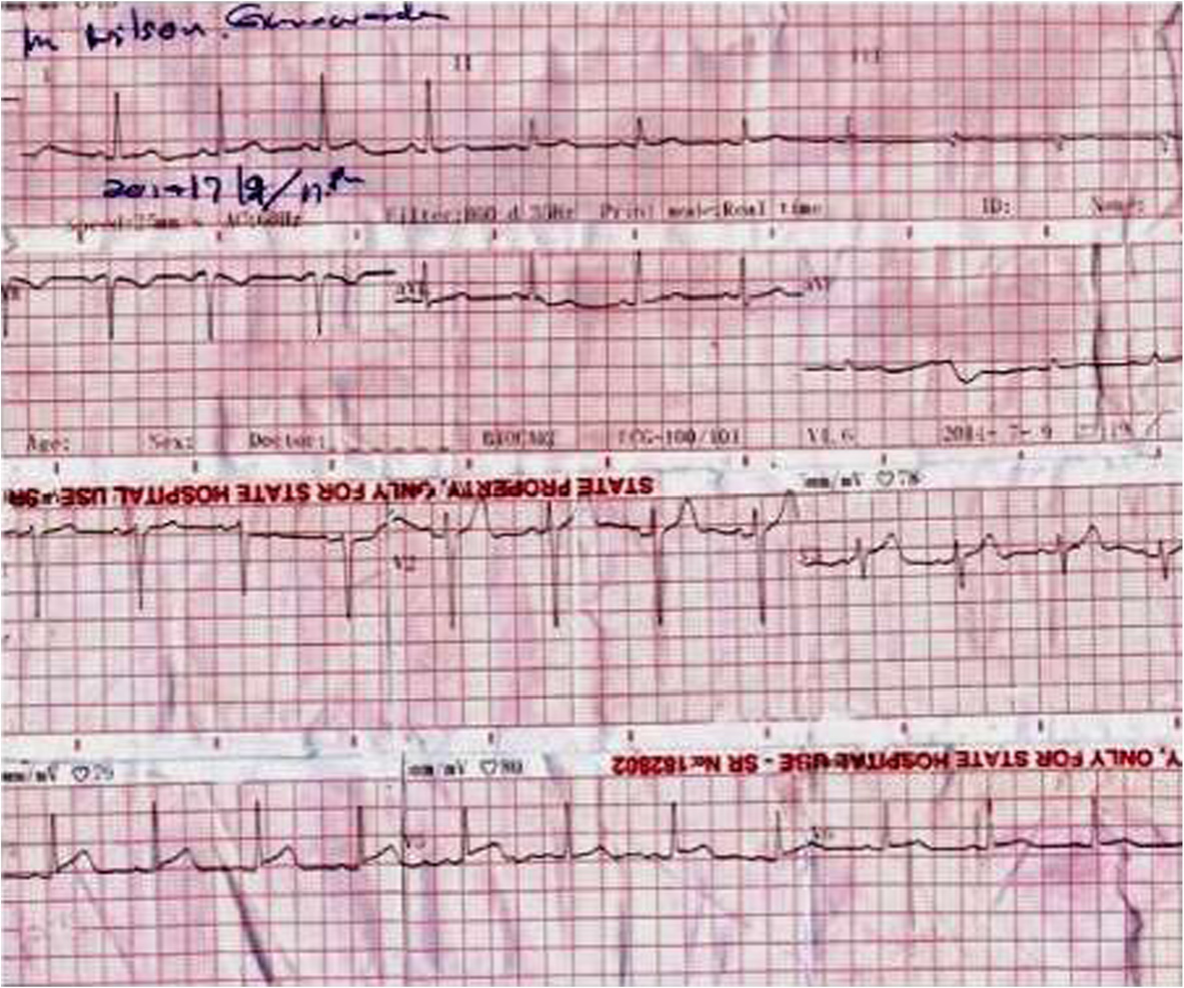
**Electrocardiogram taken at 23.00 hours showing completely resolved ST segment elevations in the inferior leads.**

**Figure 4:**
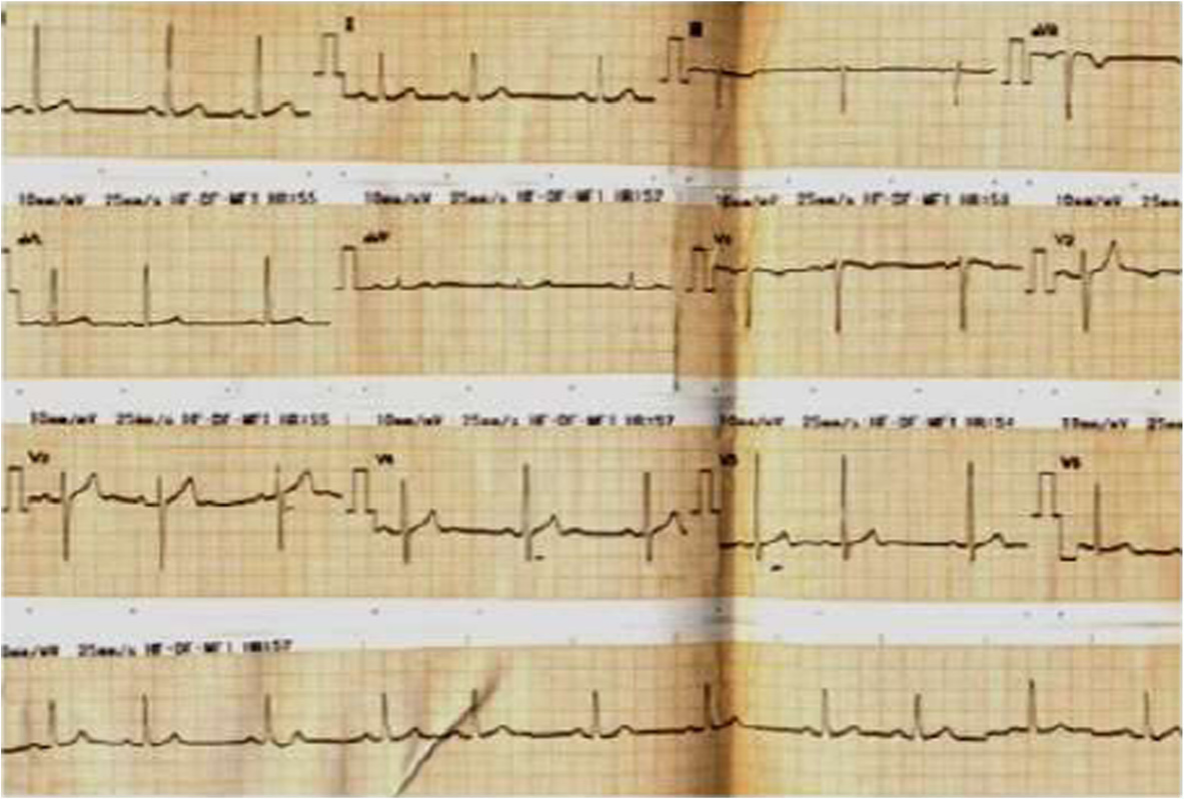
**Electrocardiogram taken the next day at 05.00 hours showing completely normalized ST segment elevations.**

As ECG showed evidence of ST elevation ACS, the patient was urgently transferred to the nearest tertiary hospital with coronary care unit(CCU) facilities.On admission to the CCU patients BP was 108/82 mmHg, pulse rate was 86/min, SaO2 96%in room air. He did not have chest pain. The ECG was taken on admission to the CCU (Figure 2) revealed settling of ST elevations in Lead 11, Lead 111, aVF and V3-V6.The patient was managed with oxygen, heparin 1000 u/hour IV, aspirin 150 mg once a day(o.d), clopidogrel 75 mg o.d, atorvastatin 20 mg o.d, metoprolol 12.5 mg o.d and tolbutamide 500 mg three times a day. Echocardiography revealed no motility disorders or regional wall motion abnormalities but there was mild concentric left ventricular hypertrophy and mild diastolic dysfunction. The repeat ECG (Figure 3) revealed further resolution of ST segment elevation. Patient was closely monitored over the next few hours and he did not develop further chest pain or complications. His troponin I, 6 hours after the onset of chest pain was 2.2 ng/ml. The patient was managed for allergic myocardial infarction (Kounis syndrome) and continued on ciprofloxacin 200 mg IV twice a day for urinary tract infection. The ECG taken the following day morning (Figure 4) showed completely resolved ST segments elevation. He improved considerably over the next few days and the repeat C-reactive protein (CRP) and white blood cells (WBC) done on third day of illness was 56 mg/dl and 8.60×10^9^/l respectively. The patient refused to undergo coronary arteries angiography and was discharged on day five of hospital admission.

## Discussion

Kounis syndrome was first described in 1991 by Kounis and Zafras as “the coincidental occurrence of chest pain and allergic reactions accompanied by clinical and laboratory findings of classical angina pectoris caused by inflammatory mediators released during an allergic insult [4]. In addition to coronary arterial involvement, the entity ”Kounis syndrome” today encompasses other arterial systems with similar physiology such as mesenteric and cerebral circulation resulting in ischaemia/infarction of the vital organs [5,6].

There are several causes that have been reported as capable of inducing Kounis syndrome. These include a number of drugs(antibiotics, analgesics, antineoplastics, contrast media, corticosteroids ,intravenous anaesthetics, non steroid anti inflammatory drugs, skin disinfectants, thrombolytics, anticoagulants, proton pump inhibitors),various conditions (angio-oedema, bronchial asthma, urticaria, food allergy, exercise induced allergy, mastocytosis, serum sickness),and environmental exposure (stings by ants, bees, wasps, jellyfish, grass cutting, millet allergy, poison ivy, latex contrast, shell fish eating, viper venom envenoming) [7].

Pathophysiologically, Kounis syndrome typically results from mast cells degranulation in the setting of an allergic insult with the subsequent release of numerous inflammatory mediators such as histamine, neural proteases, arachidonic acid products, platelet activating factors and variety of cytokines [5,8]. These chemical mediators have been implicated in coronary vasospasm and atheromatous plaque rupture leading to acute coronary syndrome. Released mediators can be preformed(histamine, neutral proteases-chymase and tryptase, platelet activating factor) or newly synthesized (cytokines,chemokines,arachidonic acid products-leukotrienes, prostaglandins) [9].They can act either locally and/or systemically, and play important roles in the activation and interaction between other cells involved in allergic reactions(macrophages, T-lympocytes, endothelial cells).The most important among the various madiators are histamine,serotonin, and leukotrienes [5].

There are three variants of Kounis syndrome. Type I variant includes patients with normal or nearly normal coronary arteries without predisposing factors for coronary artery disease in whom acute allergic attacks can induce either coronary artery spasm alone without raised cardiac enzymes and troponins [2,10,11]. Type II variant includes patients with culprit but quiescent pre-existing atheromatous disease in whom the acute allergic attacks can induce either coronary artery spasm, or plaque rupture manifesting as acute myocardial infarction [2,10]. Recently described type III variant includes patients with stent thrombosis in whom thrombus harvesting and staining with hematoxycillin-eosin and Giemsa shows the presence of eosinophils and mast cells respectively, in the pathology specimens. Furthermore, type III variant is diagnosed in patients with stent implantation who died suddenly and histological examination of coronary intima, media or adventitia adjacent to stent was found to be infiltrated by eosinophils and/or mast cells [1,2,10]. However, in all these types, the prognosis depended on the magnitude of the initial allergic response, the patient’s sensitivity, co-morbidities, the site of antibody antigen reaction, the allergen concentration and the route of allergen entrance [1,2]. Our patient was 74 years old and had type 2 diabetes mellitus and dyslipidaemia as a predisposing factor for coronary artery disease. He had no previous history of angina and his 2 echocardiograms were normal. He was unwilling to undergo exercise stress testing and coronary angiography. But without coronary angiogram it was difficult to determine which type of Kounis syndrome he actually had.

It is a known fact that adrenaline can accelerate thrombus formation in animals and in man possibly by increased factor v activity and shown in animals to release thromboplastin-like substance from the walls of blood vessels [12,13]. It causes both coronary vasodilatation and myocardial oxygen demand by direct ionotropic and chronotropic effects. Adrenaline has been used historically as a provocation test for angina pectoris and often used in the treatment of anaphylactic shock [13]. Though adrenaline is a life saving drug in anaphylaxsis it can cause adverse effects in sulfite allergic patients. Epinephrine(Adrenaline) contains metabisulfite as a preservative [14]. Anaphylactoid shock from ehinephrine-containning metasulfite occurred during epidural anaesthesia for cessarian section [15]. There are reports of hypersensitivity, anaphylaxis and even death from Kounis syndrome from sulfite administration [3,14]. Sulfites have a useful role to play in helping preserve many foods and beverages. Having consumed such foods or drinks people may have experienced allergic conditions in the past. So it is very important to take detailed allergic history including history of sulfite allergy when considering the use of adrenaline. Adrenaline is still the primary drug for anaphylaxis, but avoidance of medications containing sulfites should be considered in the sulfite-sensitized patient [14]. Preservative-free epinephrine is now available. It should be given intramuscularly (IM) because it has a faster onset of action and maintains a more stable concentration compared to the subcutaneous route [recommended IM dose,0.2-0.5 mg(1:1000)] [14].

Our patient’s chest pain occurred about 20 minutes after the first dose of IV antibiotic administration but before adrenalin was given. Also his ECG changes appeared before giving adrenalin. This evidence supports the occurrence of this acute coronary event as a direct consequence of the co- amoxyclavulanic acid. On the other hand, hypotension caused by anaphylaxis can lead to myocardial hypoperfusion and acute ischemia. Even though this patient recovered from anaphylactic shock within a few minutes of emergency treatment, it took several hours for ECG changes to reverse. It may be possibly due to the prolonged effects of anaphylaxis and released mediators for myocardium and coronary vasculature.

With regard to the therapeutic approach to coronary spasms following an allergic reaction, the medications should include vasodilators, such as nitrates, and calcium channel inhibitors, which are in any case the treatment of choice for every case of coronary spasm [1,2,16]. In contrast, the role of corticosteroids and antihistamines, apart from their clear usefulness in the treatment of systemic manifestations of allergy has not been fully determined. In other words, it is not known to what extent these, and other pharmaceutical agents have a stabilizing action on the membrane of mast cells or restrict the action of mediators of the allergy, or play a role in the treatment of acute coronary events that are caused by allergic reactions [2,16]. Drugs including mediator antagonists, inhibitors of mediator biosynthesis, leukotriene antagonists, mediator receptor blockers such as sodium nedocromil, sodium cromoglycate, ketotifen, humanized IgG1 monoclonal antibodies and others which interfere with mast cell stabilization and prevent the release of mast cell contents and could emerge as novel therapeutic modalities capable of preventing acute coronary syndrome [1,2,10].

## Conclusion

Our patient suffered from amoxicillin-clavulanic acid induced hypersensitivity reaction, leading to hypersensitivity associated ACS or Kounis syndrome. This case highlights the need for physicians to be aware of the allergic myocardial infarction. The diagnosis of Kounis syndrome should be entertained when acute onset chest pain is accompanied by allergic symptoms, electrocardiographic changes and elevated cardiac enzymes. Kounis syndrome has emerged as a life-threatening condition irrespective of aetiology of the allergic reaction.. In cases of hypersensitive reactions, the ECG has become an essential investigation. Future studies are needed to understand the mechanisms of allergy causing acute coronary syndromes as this will pave way for effective therapeutic interventions.

## Consent

Written informed consent was obtained from the patient for publication of this Case Report and accompanying images. A copy of the written consent is available for review by the Editor-in-Chief of this journal.

## Abbreviations

ECG: Electrocardiogram
ETU: Emergency Treatment Unit
2D: Two dimensional
hpf: High power field
RBC: Red blood cells
WBC: White blood cells
CRP: C-reactive proteins
im: Intramuscular
iv: Intravenous
BP: Blood pressure
o.d: Once per day
tds: Three times per day.
